# A Novel Ferroptosis-Related Gene Prognosis Signature and Identifying Atorvastatin as a Potential Therapeutic Agent for Hepatocellular Carcinoma

**DOI:** 10.3390/cimb47030201

**Published:** 2025-03-18

**Authors:** Ling Wang, Xiaoqin He, Yang Shen, Jiayu Chen, Yukai Chen, Zhuolin Zhou, Ximing Xu

**Affiliations:** Cancer Center, Renmin Hospital of Wuhan University, Wuhan 430060, China; wangling@whu.edu.cn (L.W.); 2014203020097@whu.edu.cn (X.H.); 2017302180196@whu.edu.cn (Y.S.); 2015283020224@whu.edu.cn (J.C.); 2017302180083@whu.edu.cn (Y.C.); 2018305231023@whu.edu.cn (Z.Z.)

**Keywords:** ferroptosis, FRG signature, prognosis, hepatocellular carcinoma, CMap database, antitumor drug

## Abstract

Among the most common malignant tumors, hepatocellular carcinoma (HCC) is a primary liver cancer type that has a high mortality rate. HCC often presents insidiously, is prone to recurrence, and has limited treatment efficacy. Ferroptosis regulates tumorigenesis, progression, and metastasis, which is a novel form of iron-dependent cell death. Numerous studies suggest that HCC is sensitive to ferroptosis, indicating that targeted therapies aimed at inducing ferroptosis may represent a promising new approach to cancer treatment. This study aims to find genes associated with HCC and ferroptosis, as well as to screen for potential agents that may cause ferroptosis in HCC. Transcriptome and clinical sample data were obtained from the TCGA database to identify differentially expressed genes related to ferroptosis. Using various regression and survival analysis techniques, we developed a prognostic model based on four core genes and evaluated its predictive potential. Subsequently, we screened for potential therapeutic agents in the Connective Map (CMap) database, designated as compound Atorvastatin, based on differential genes from two risk groups and related to ferroptosis. Through experiments conducted in vivo and in vitro, we demonstrated that Atorvastatin can induce ferroptosis in HCC cells while inhibiting their growth and migration. In conclusion, this research targets ferroptosis therapy and provides new insights for improving the prediction and prevention of HCC.

## 1. Introduction

Hepatocellular carcinoma (HCC) is one of the most prevalent malignant tumors globally, accounting for 75%~85% of primary liver cancers, and is characterized by high rates of metastasis, recurrence, and mortality. In recent years, its incidence has continued to rise globally, particularly sharp increases observed in Asia [1,2]. Studies indicate that hepatitis B and C virus infections, chronic alcohol consumption, metabolic disorders, and exposure to aflatoxin B1 are major risk factors for liver cancer [3]. Liver resection, ablation, and transplantation offer potential curative options; however, successful outcomes depend on diagnosis at an adequately early stage [4]. HCC is often diagnosed at advanced stages, resulting in the majority of patients missing the optimal treatment window. Furthermore, failure to identify patients at high risk for metastasis and recurrence contributes to poor prognoses; data indicate that nearly 70% of individuals have a recurrence within five years following surgery [5]. Therefore, there is a pressing need to develop more efficient biomarkers for the early detection and prognostic prediction of HCC [6]. Recent reports have emphasized the considerable potential of genes associated with ferroptosis in both the treatment and prediction of prognosis in HCC, suggesting possibilities for early diagnosis and personalized therapy for patients [7,8,9].

Ferroptosis is a form of programmed cell death characterized by the disruption of redox homeostasis. The chelation of excess iron and the activation of glutathione peroxidase 4 (GPX4)-dependent and -independent antioxidant pathways serve as inhibitory mechanisms against ferroptosis [10]. Extensive research indicates that ferroptosis plays a critical role in tumor suppression, thereby providing new opportunities for cancer prevention, diagnosis, and treatment [11]. Studies have reported that SLC7A11 and GPX4 act as negative regulators of ferroptosis. Compared to normal liver tissue, both are upregulated in HCC and exhibit lower levels of ferroptosis [12]. *MT1* is a ferroptosis-related gene capable of participating in the induction of ferroptosis in HCC [13]. Targeting ferroptosis has been identified as an effective and promising strategy for anticancer therapy. A substantial number of preclinical studies and clinical trials on ferroptosis have been conducted [14,15], with ferroptosis inducers such as sulfasalazine, artemisinin, and sorafenib approved by U.S. Food and Drug Administration (FDA) as anticancer agents for cancer treatment. Therefore, this study is grounded in the foundational context of ferroptosis-based cancer therapy and aims to establish prognostic features of ferroptosis-related genes using bioinformatic approaches, as well as to search for novel therapeutic compounds for HCC, ultimately leading to experimental validation.

In our study, we first downloaded the mRNA expression profiles and clinical data of HCC patients from The Cancer Genome Atlas (TCGA) and the International Cancer Genome Consortium (ICGC) to construct a prognostic model based on ferroptosis-related differential genes in HCC. Subsequently, we screened for potential anticancer agents, specifically Atorvastatin, from the Connectivity Map (CMap) database and demonstrated its role in inducing ferroptosis in HCC. This research provides new directions for the diagnosis and treatment of HCC, potentially enhancing survival rates through personalized therapy, and further strengthens the theoretical basis for the function of ferroptosis in the prognostic prediction and treatment of HCC.

## 2. Materials and Methods

### 2.1. Cell Culture and Reagents

Procell Life Science & Technology Co., Ltd. (Wuhan, China) provided Human HCC cell lines (MHCC-97H and Huh7). The cells were cultured in Dulbecco’s modified Eagle’s medium (DMEM) fixed with 10% FBS (Gibco, Grand Island, NY, USA) at a 37 °C, 5% CO2 incubator.

Atorvastatin (purity ≥ 98%), Ferrostatin-1 (Fer-1, purity ≥ 98%), and dimethyl sulfoxide (DMSO) were provided by APExBIO Technology LLC (Houston, TX, USA). DMSO was used to dissolve Atorvastatin, and, then, it remained at −80 °C.

This study used the following antibodies: GAPDH, GPX4, xCT, and Ferritin antibodies, which were sourced from Abmart (Wuhan, China).

### 2.2. Data Source

The mRNA expression data and relevant clinical information of HCC from TCGA (http://www.cancergenome.nih.gov/, accessed on 7 March 2024) were obtained as an analysis dataset. This dataset included 374 HCC cancer samples and 50 normal samples. Liver Cancer—RIKEN, JP Project from ICGC (https://dcc.icgc.org/releases/current/Projects, accessed on 10 March 2024)—transcriptomic data were downloaded as a validation cohort, including 273 HCC samples and 203 normal samples. Additionally, expression profile data of 247 HCC patients were obtained from the Gene Expression Omnibus (GEO) database (GSE14520, https://www.ncbi.nlm.nih.gov/geo, accessed on 3 April 2024). Relevant ferroptosis genes were extracted from the FerrDb database (http://www.zhounan.org/ferrdb/index.html, accessed on 7 March 2024), and 484 ferroptosis-related genes (FRGs) were finally acquired for study by removing the duplicate data from the combined dataset, which are provided in Supplementary Table S1. Immunohistochemical data of several FRGs in HCC tissues and normal liver tissues were obtained from the online Human Protein Atlas (HPA) database (https://www.proteinatlas.org/, accessed on 15 May 2024). Single-cell RNA-seq data were downloaded from the Human Liver Browse database (Human Liver Browser (weizmann.ac.il, accessed on 15 May 2024)) [16].

### 2.3. Extraction of Differentially Expressed Ferroptosis-Related Genes

Using the “limma” R package, a differential expression analysis of liver tumor and normal samples in the TCGA cohort was conducted with the selected filtering criteria (|log2FC| > 1 and adjusted *p* < 0.05). The results were then intersected with FRGs to identify differentially expressed genes associated with ferroptosis (FDEGs).

### 2.4. Identification and Validation of a Prognostic Model for HCC Associated with Ferroptosis

Univariate Cox regression analysis was carried out using the “survival” R package to evaluate the connection between FDEGs and survival in the TCGA cohort, yielding *p*-values and hazard ratios (HRs) for each gene. Genes with *p* < 0.05 were deemed to be significantly associated with patient prognosis. Higher HR values indicate a greater risk associated with the gene. Through the utilization of LASSO regression analysis on the TCGA training set, a definitive set of four FDEGs was identified for the construction of the prognostic risk model. The standardized expression levels of four FDEGs and their respective regression coefficients were multiplied to calculate the risk score for HCC patients. The formulation can be expressed through the following equation:

$$Risk\;Score=\sum_{i=1}^{n}\left(Coef\_i\times x\_i\right)$$

Using this method, risk scores were computed for both the TCGA and ICGC cohorts. The median risk score was used as the criterion to separate patients in each cohort into high-risk and low-risk groups. Subsequently, univariate and multivariate Cox regression analyses were conducted to assess whether the risk score model demonstrated robust prognostic capacity independent of other clinicopathological characteristics, including age, sex, and stages. The Receiver Operating Characteristic (ROC) curve can be used to evaluate the ability of the risk score to diagnose or predict patient overall survival (OS), based on sensitivity and specificity. Prognostic predictions for 1-year, 2-year, and 3-year OS were generated in both datasets, with subsequent calculation of the area under the curve (AUC).

### 2.5. Functional Enrichment Analysis

Based on differentially expressed genes in the two risk groups, Kyoto Encyclopedia of Genes and Genomes (KEGG) and Gene Ontology (GO) analyses were conducted with the “clusterProfiler” and “enrichplot” R packages. The results were deemed significant when *p* < 0.05.

### 2.6. Screening for Potential Therapeutic Agents in the Database

The CMap database was utilized to identify compounds associated with specific input gene signatures. Differential risk analysis of the TCGA dataset revealed differentially expressed genes between high- and low-risk groups. These genes along with FRGs (Table S3) were independently queried in the CMap database (https://clue.io/query, accessed on 17 May 2024) to obtain two compound groups. A screening threshold of adjusted *p*-value > 1.5 combined with scores < −1 was applied for candidate drug selection. Subsequent intersection analysis of the two compound groups identified core potential drugs that may target ferroptosis for HCC treatment.

### 2.7. Detection of Cell Migration and Activity

The CCK-8 test (Servicebio, Wuhan, China) was used to estimate cell viability. Cells were seeded into 96-well plates at a density of 8000 cells/well. Then, they were treated with different concentrations of Atorvastatin (0, 0.5, 1, 2, 4, 6, 8, 10, 12, and 14 μM) and cultured for 24 h. Each well was filled with 10 µL of CCK-8 solution and remained in the incubator for 2 h, followed by measuring the absorbance at 450 nm. The cell viability was calculated using the following formula: Cell viability (%) = [A(treatment) − A(blank)]/[A(control) − A(blank)].

To perform the wound healing assay, cells were cultured in six-well plates until reaching an appropriate confluence. A straight line was drawn by a sterile 200 µL pipette tip on the cell layer. The cells were subsequently cultured in a serum-free medium. Images were taken at 0 and 48 h after the scratching, and the wound area was measured and analyzed using ImageJ (V1.54f) software to assess the degree of cell migration.

The Transwell assay was used to measure the invasion of HCC cells. Matrigel was diluted in serum-free medium at a ratio of 1:8 and applied to the Transwell membrane, followed by incubation at 37 °C for 3 h. Serum-free cell suspension was added to the inner chamber, while 20% serum was added to the outer chamber. The cells in the chamber were washed, fixed, and stained after 24 h, and, then, they were counted in three different fields of view under a microscope.

### 2.8. Intracellular Reactive Oxygen Species Detection

HCC cells were divided into four groups: negative control (NC), Atorvastatin treatment (A), Fer-1, and combination treatment (Fer-1 + A). After 24 h of drug treatment for determination of ROS levels, each group was treated with 10 μM of 2′,7′-Dichlorodihydrofluorescein (DCFH-DA) (Beyotime, Wuhan, China) following the product manuals. The fluorescence intensity was detected by flow cytometry and observed under a fluorescence microscope.

### 2.9. Western Blotting Analysis

Total protein was extracted from HCC cells and quantified using a BCA protein assay kit (Beyotime, Wuhan, China). Proteins were separated by sodium dodecyl sulfate-polyacrylamide gel electrophoresis (SDS-PAGE) and subsequently transferred to a polyvinylidene fluoride (PVDF, Millipore, NJ, USA) membrane. After blocking the membrane with 5% skim milk powder for 1 h, it was incubated overnight at 4 °C with the corresponding primary antibody, followed by a 1 h incubation at room temperature with the secondary antibody. Finally, immunoreactive protein bands were visualized using an ECL imaging system (ChemiDoc, Bio-Rad, Hercules, CA, USA). Band intensity was quantified using ImageJ software, and the X protein to GAPDH band intensity ratio was used to express the results.

### 2.10. In Vivo Xenograft Model

Male *BALB/c nude mice* aged five weeks were obtained from Wuhan Moubaili Biotechnology Co., Ltd. (Wuhan, China) and housed in a dedicated facility at the Animal Experiment Center of Renmin Hospital, Wuhan University. This study was approved by the Ethics Committee (Ethics Code: WDRM 202300384) and conducted following the center’s standard guidelines.

MHCC-97H cells were resuspended in PBS (1 × 10^7^/mL) and subsequently subcutaneously implanted into the dorsal region of BALB/c nude mice. Four groups of nude mice were randomly separated (three mice/group) and administered equivalent volumes of DMSO, Atorvastatin (10 mg/kg), Fer-1 (1 mg/kg), and Fer-1 (1 mg/kg) + Atorvastatin (10 mg/kg) via intraperitoneal injection every two days. Tumor growth was monitored daily. The mice were dissected for further study after receiving tumor cell injections for four weeks.

### 2.11. Statistical Analysis

The analysis was conducted using SPSS (V.26), GraphPad Prism (V9.0), Image J (V1.54f), and R (V4.2.3) software. An independent t-test was utilized for comparing two sample groups, while one-way ANOVA was employed for comparisons among multiple groups. Results for measurement data are presented as the mean ± standard deviation (SD). The prognostic model’s predictive performance was assessed through the Kaplan–Meier method and the log-rank test, with statistical significance defined as *p* < 0.05.

## 3. Results

### 3.1. Characteristics of Hepatocellular Carcinoma Datasets from TCGA and ICGC

This study obtained data on 424 cases from the TCGA database, which included 50 normal datasets and 374 tumor datasets. After excluding cases with a survival period of less than 30 days and incomplete clinical data, a total of 326 cases met the inclusion criteria. Additionally, we downloaded 445 cases from the ICGC database, which included 258 cases with available clinical characteristics for analysis. Detailed clinical information on the included cases, including age, sex, survival time and status, is presented in Table 1.

### 3.2. Identification of Ferroptosis-Associated Differential Genes in the TCGA Cohort

**Figure 1:**
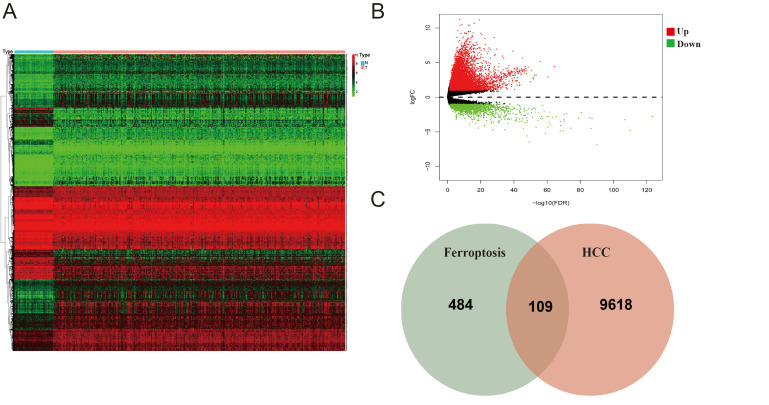
Identification of differentially expressed ferroptosis-related genes in the TCGA cohort: (**A**) The heatmap of differentially expressed genes in the TCGA cohort. (**B**) Volcanic plots of gene expression of DEGs. (**C**) Venn diagram of ferroptosis-related DEGs.

Differential gene expression analysis was conducted on 374 HCC samples and 50 normal liver tissue samples. Using *p* < 0.05 and |log2 FC| > 1 as selection criteria, we identified 9618 differentially expressed genes (DEGs)between tumor and normal tissues, of which 8031 were upregulated and 1587 were downregulated. They were further analyzed by clustering, as illustrated in the volcano plot and heatmap (Figure 1A,B).

Through searching in the FerrDb database, we discovered 484 ferroptosis-related target genes (Table S1). By intersecting DEGs with the ferroptosis-related genes, we identified 109 overlapping genes (69 upregulated and 40 downregulated), which were used for subsequent analyses (Figure 1C).

### 3.3. Identification of Prognostic Ferroptosis-Related Genes in the TCGA Cohort

**Figure 2:**
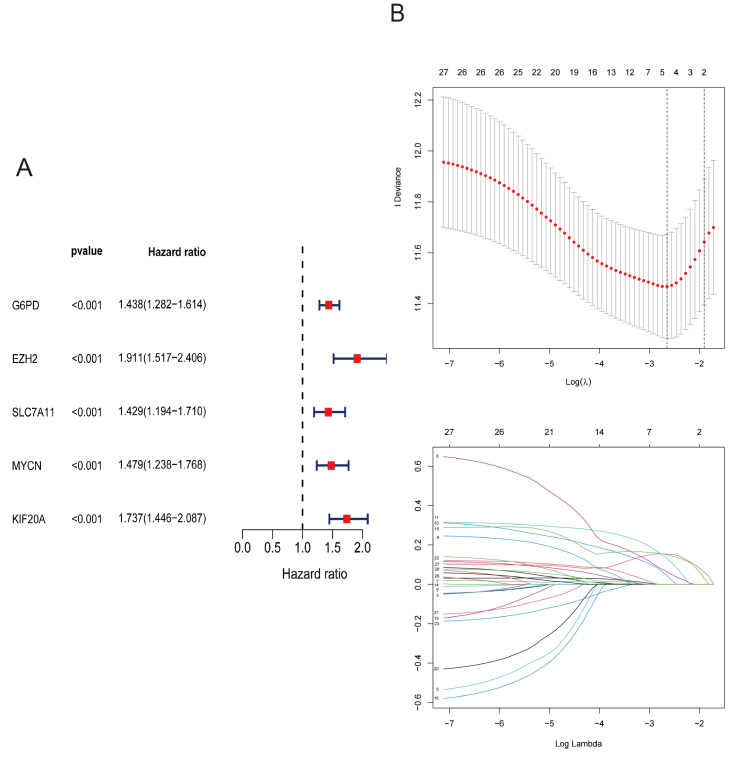
Identification of prognostic ferroptosis-related genes in the TCGA cohort: (**A**) The forest plot of the univariate Cox. (**B**) The cross-validation fit plot of LASSO Cox analysis.

By integrating the overlapping genes with clinical data on patient survival status and OS from the TCGA cohort, univariate Cox analysis, and Lasso regression identified five ferroptosis-related prognostic genes as key genes for further analysis (Figure 2A,B).

### 3.4. Construction of Risk Score in the TCGA Cohort

**Figure 3:**
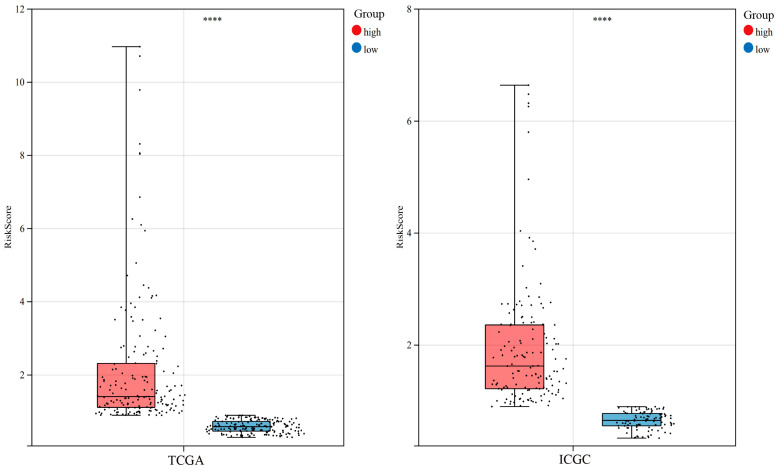
Distribution of risk scores in TCGA and ICGC cohorts. **** *p* < 0.0001

The Akaike Information Criterion (AIC) is a statistic used for model selection, balancing model complexity and goodness of fit by minimizing the AIC value. For the five FDEGs identified in the previous analysis, we optimized the model further by selecting the optimal AIC value, ultimately excluding *EZH2* and obtaining four genes with associated coefficients. The risk score = (0.175160337 × Exp*G6PD*) + (0.226116013 × Exp*SLC7A11*) + (0.325445288 × Exp*MYCN*) + (0.326570746 × Exp*KIF20A*). For each patient in the TCGA cohort and ICGC cohort, we calculated risk scores using the above formula and stratified them into low- and high-risk groups according to the median score. The distribution of risk scores in high- and low-risk groups in the data cohorts is visualized through box plots (Table S2, Figure 3).

### 3.5. Verification of the Prognostic Model of the TCGA Cohort Based on the ICGC Cohort

**Figure 4:**
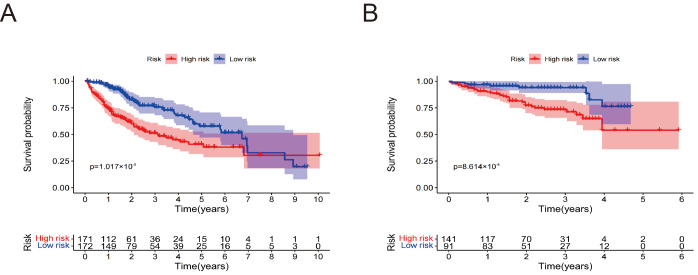

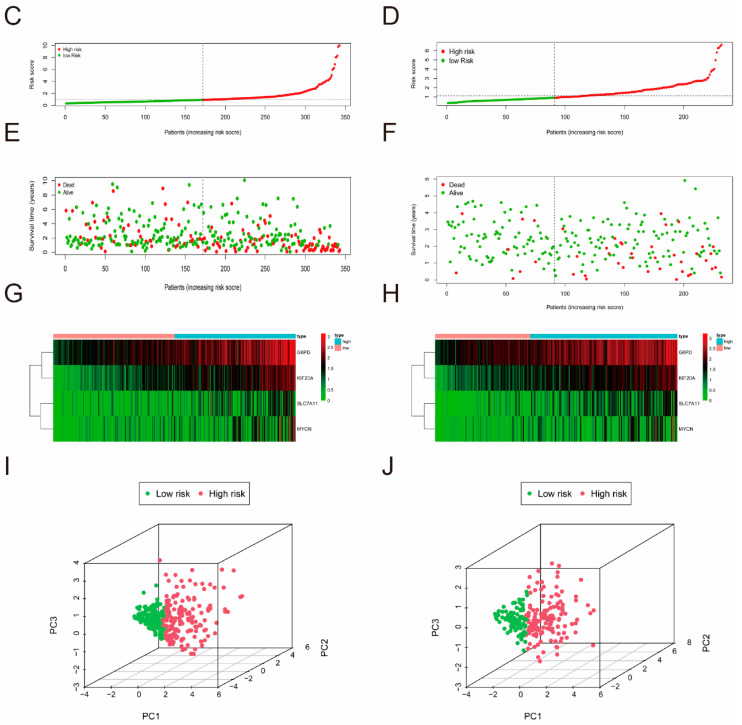
Verification of the prognostic model based on the ICGC cohort. The Kaplan-Meier curve of the TCGA cohort (**A**) and ICGC cohort (**B**). The risk score distribution of the TCGA cohort (**C**) and ICGC cohort (**D**). The survival status distribution of TCGA cohort (**E**) and ICGC cohort (**F**). Heatmap of four crucial FDEGs of TCGA cohort (**G**) and ICGC cohort (**H**). PCA plot of TCGA cohort (**I**) and ICGC cohort (**J**).

The Kaplan-Meier survival curve was used to assess the model’s effectiveness in predicting OS, revealing that HCC patients with low risk had significantly improved OS compared to those with high risk in the TCGA (Figure 4A) and ICGC cohorts (Figure 4B). In addition, in both training and validation cohorts, the risk score increased from left to right (Figure 4C,D). It was observed that there was an increase in the number of high-risk patients and deaths in correlation with the risk score (Figure 4E,F). Additionally, the heat maps show that, in both cohorts, the expression of *G6PD*, *SLC7A11*, *MYCN*, and *KIF20A* increases with rising risk scores (Figure 4G,H). PCA plots showed a significant separation between the two risk groups (Figure 4I,J). These findings demonstrate that the prognostic model exhibits comparable predictive performance in the ICGC cohort to that observed in the training cohort. The risk score may serve as a prognostic indicator for HCC patients and effectively predicts OS.

### 3.6. Risk Score as an Independent Predictor in HCC

**Figure 5:**
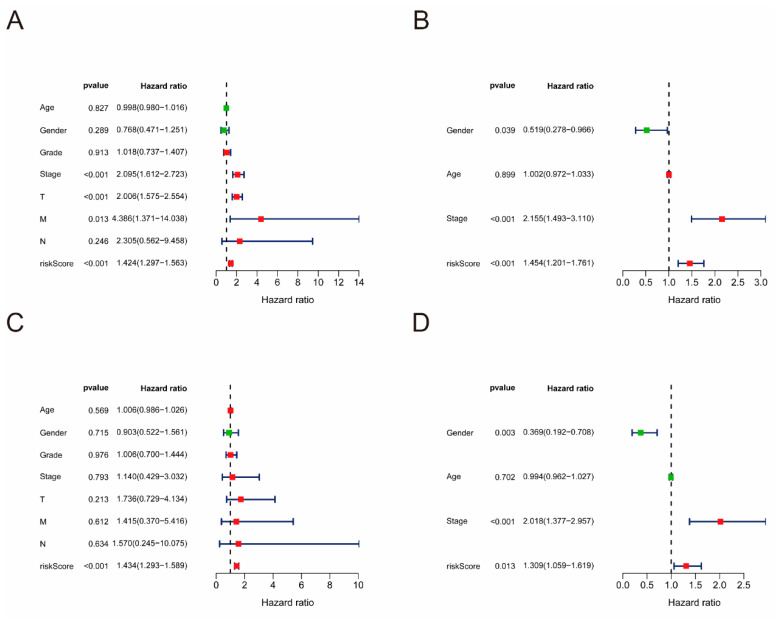
Univariate and multivariate Cox regression analyses in HCC. Forest plot of the univariate Cox regression analysis of TCGA cohort (**A**) and ICGC cohort (**B**). Forest plot of the multivariate Cox regression analysis of TCGA cohort (**C**) and ICGC cohort (**D**).

We employed both univariate and multivariate Cox regression analyses to assess whether the predictive risk scores in the TCGA and ICGC cohorts were independent of clinical data. Univariate Cox regression analysis revealed a significant association between risk scores and OS in both the TCGA and ICGC cohorts (HR = 1.424, *p* < 0.001; HR = 1.454, *p* < 0.001). The occurrence of poor survival results increases with the elevation of risk scores (Figure 5A,B). In addition, the analysis also demonstrated that stage and T staging were statistically different. The multivariate Cox regression analysis showed that the risk score functions as an independent predictive indicator of OS in patients with HCC after adjusting for other complicated factors including age, gender, grade, and TNM stage (HR = 1.434, *p* < 0.001; HR = 1.309, *p* = 0.013) (Figure 5C,D).

### 3.7. Receiver Operating Characteristic Curve Analysis

**Figure 6:**
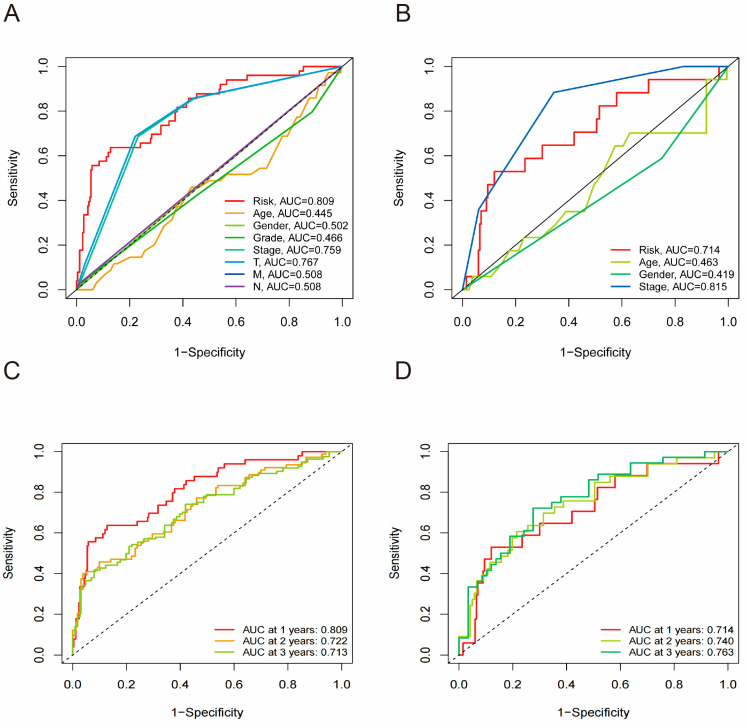
The ROC curve of TCGA and ICGC cohorts. The ROC curve of TCGA cohort (**A**) and ICGC cohort (**B**). The AUC of time-dependent ROC curves in the TCGA cohort (**C**) and ICGC cohort (**D**).

We used ROC analysis to assess the predictive accuracy of the risk scores derived from the prognostic model for long-term survival (Figure 6A,B). In the TCGA cohort, the risk score demonstrated AUC values of 0.809, 0.722, and 0.713 for the 1-year, 2-year, and 3-year time-dependent ROC curves, respectively (Figure 6C). In the ICGC cohort, the AUC for risk scores at 1, 2, and 3 years was 0.714, 0.740, and 0.763, respectively (Figure 6D). Thus, this predictive model is independent of other clinical parameters and can serve as a novel and reliable prognostic factor for patient outcomes.

### 3.8. The Expression Levels of Core Genes and GSEA Enrichment Analysis of the Risk Score

**Figure 7:**
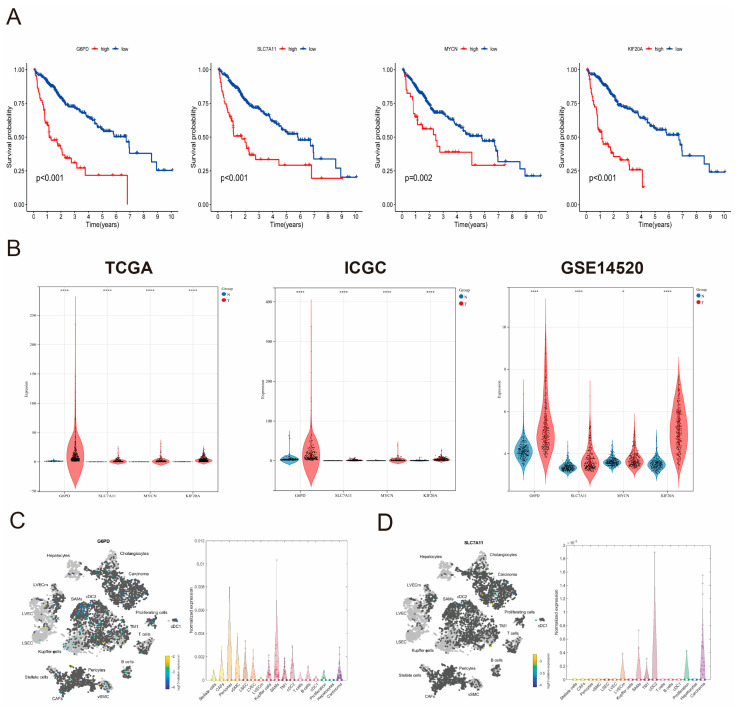

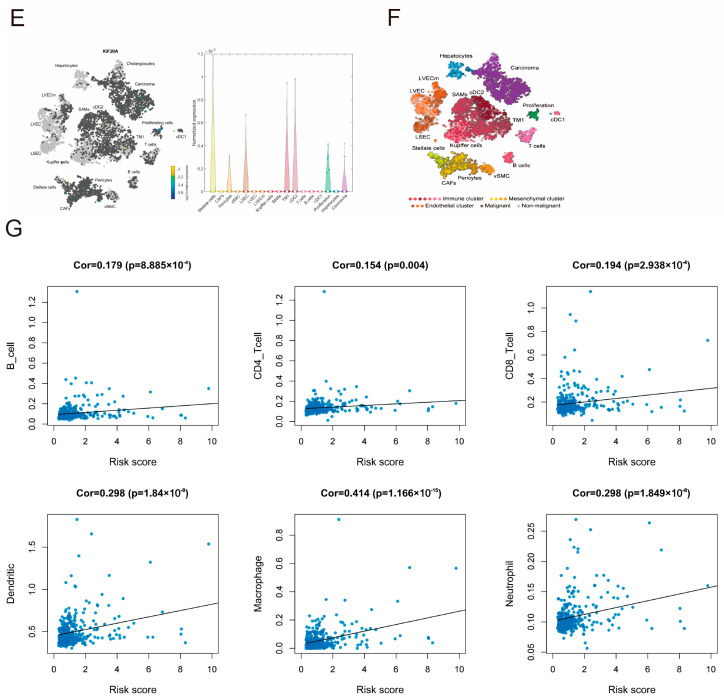

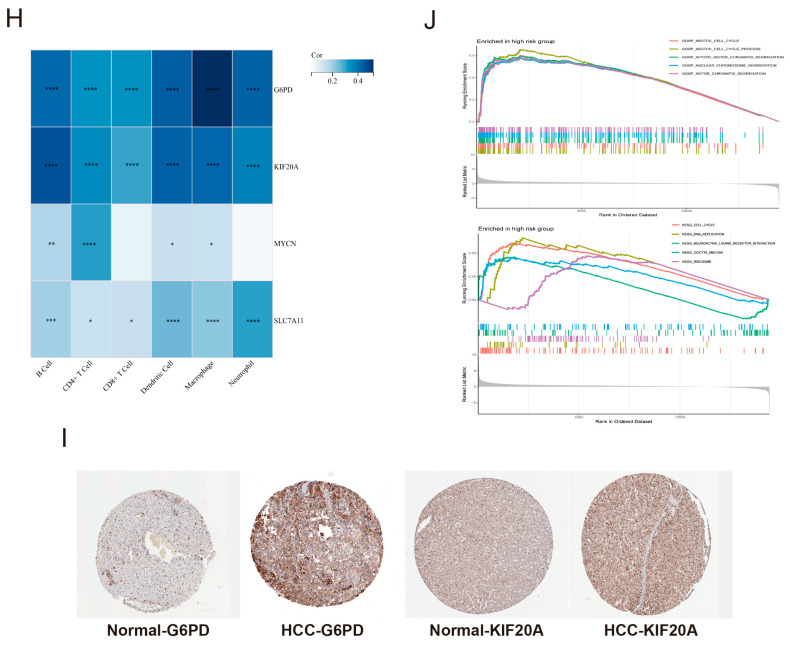
Expression levels of four genes and GSEA enrichment analysis of the risk score. (**A**) The Kaplan-Meier curves of four genes. (**B**) Upregulated expression of four genes in the TCGA, ICGC, and GSE14520 data. (**C**) Increased expression and distribution of G6PD, (**D**) SLC7A11, and (**E**) KIF20A in single-cell RNA-seq data. (**F**) Plot of normalized expression of genes in different cells from single-cell RNA-seq analysis. (**G**) Correlations between the risk score and immune cell infiltration. (**H**) Correlations between the genes and immune cell infiltration. (**I**) The protein expression levels of G6PD and KIF20A in the HPA database. (**J**) The GSEA analysis of the high-risk group. * *p* < 0.05, ** *p* < 0.01, *** *p* < 0.001, **** *p* < 0.0001.

We conducted additional validation of the four above ferroptosis-related prognostic genes. Further study showed that poor OS in HCC patients has been associated with elevated expressions of *G6PD*, *SLC7A11*, *MYCN*, and *KIF20A* (Figure 7A). Subsequently, analysis in the TCGA, ICGC, and GEO databases revealed that the expression of these genes was upregulated in HCC tissues compared to adjacent normal liver tissues (Figure 7B). The same findings were observed when single-cell RNA-seq data were analyzed. Importantly, according to the single-cell RNA-seq data, the four genes are expressed in hepatocytes as well as other cell types, including type 2 conventional dendritic cells (cDC2) and sympathetic neuron-associated macrophages (SAMs) (Figure 7C–F), suggesting potential associations between risk scores and tumor microenvironment (TME) characteristics. To further investigate the relationship between risk scores and TME features, we generated heatmaps demonstrating correlations between gene expression and immune cell infiltration, and we employed the TIMER algorithm to estimate associations between risk scores and infiltration levels of distinct immune cell populations. Significant positive correlations were identified between risk scores and infiltration densities of B cells, CD4 + T cells, CD8 + T cells, neutrophils, macrophages, and dendritic cells (Figure 7G,H). Immunohistochemistry (IHC) staining results from the HPA database further provided protein levels of G6PD and KIF20A in HCC and normal tissues, the same as findings from the TCGA data (Figure 7I). These results suggest that the four genes were correctly selected as the core genes for constructing the prognostic signature of HCC.

The GSEA analysis indicated that the high-risk group is associated with cellular division processes, including mitosis, meiosis, and chromosomal segregation. Moreover, cell cycle regulation and DNA replication-related pathways are the main regulatory pathways involved (Figure 7J). In summary, GSEA analysis suggests that the prognostic risk genes may regulate the biological processes of HCC by influencing the cell cycle.

### 3.9. Screening the Ferroptosis-Targeting Therapeutics for HCC from the CMap Database

Risk differential analysis of TCGA expression data identified 141 differentially expressed genes between high- and low-risk groups (Figure S1). Using these genes combined with the previously 109 ferroptosis-related genes, we queried the CMap database to identify potential therapeutic agents for HCC and key compounds inducing ferroptosis (Table S3). The top 10 significant compounds from each category were tabulated (Table 2 and Table 3). Following filtration criteria (adjusted *p*-value > 1.5 and scores < −1), intersecting compounds were prioritized as potential ferroptosis-targeting therapeutics, including clinically validated agents such as azacitidine and etoposide. Validation from the literature revealed that Atorvastatin, a highlighted compound, exhibits documented anti-HCC efficacy in prior studies [17,18,19], supporting the credibility of our screening strategy. While Atorvastatin demonstrates preclinical relevance in oncology, its mechanism associated with ferroptosis in HCC remains unexplored. We propose Atorvastatin as a ferroptosis-modulating therapeutic candidate for HCC, warranting further experimental validation.

### 3.10. Atorvastatin Inhibits the Proliferation and Migration of HCC Cells

**Figure 8:**
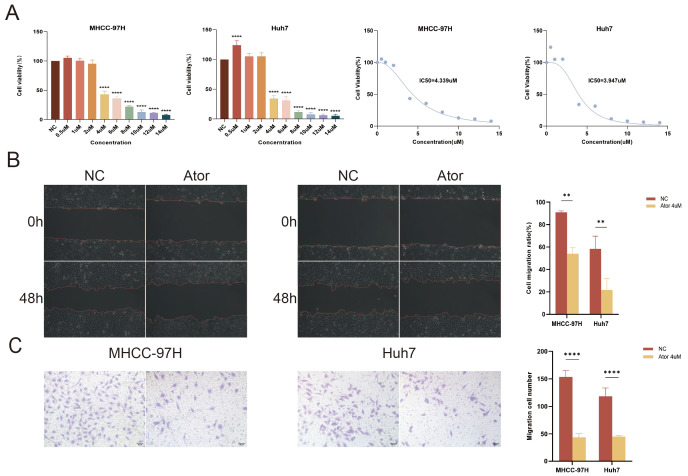
Atorvastatin inhibited the proliferation and migration of HCC cells in vitro. (**A**) Effects of different concentrations of Atorvastatin on the cell activity in MHCC-97H and Huh7. (**B**) The wound healing assay assesses the migration ability of MHCC-97H and Huh7 treated with Atorvastatin. Statistical results of wound closure are presented. Scale bar: 50 μm. (**C**) Cell invasion ability of HCC cells treated with Atorvastatin is determined by Transwell assay. Statistical results are presented. Scale bar: 50 μm. Data are presented as mean ± SD from three independent experiments. ** *p* < 0.01and **** *p* < 0.0001 compared to NC group.

After exposing two HCC cell lines to varying concentrations of Atorvastatin for 24 h, cell viability decreased following the increasing concentration of Atorvastatin. The IC50 (half-maximal inhibitory concentration) is a crucial metric for assessing the inhibitory effect of a drug on cell proliferation. We calculated the IC50 values for Atorvastatin in the MHCC-97H and Huh7 cells (4.339 μM, 3.947 μM), ultimately establishing a concentration of 4 μM for subsequent drug trials (Figure 8A). The results of the wound healing and Transwell assays demonstrate that Atorvastatin significantly inhibits the migratory and invasive capabilities of HCC cells (Figure 8B,C). These results underscore a substantial inhibition of HCC cell growth by Atorvastatin.

### 3.11. Atorvastatin Induces Ferroptosis in HCC Cells

**Figure 9:**
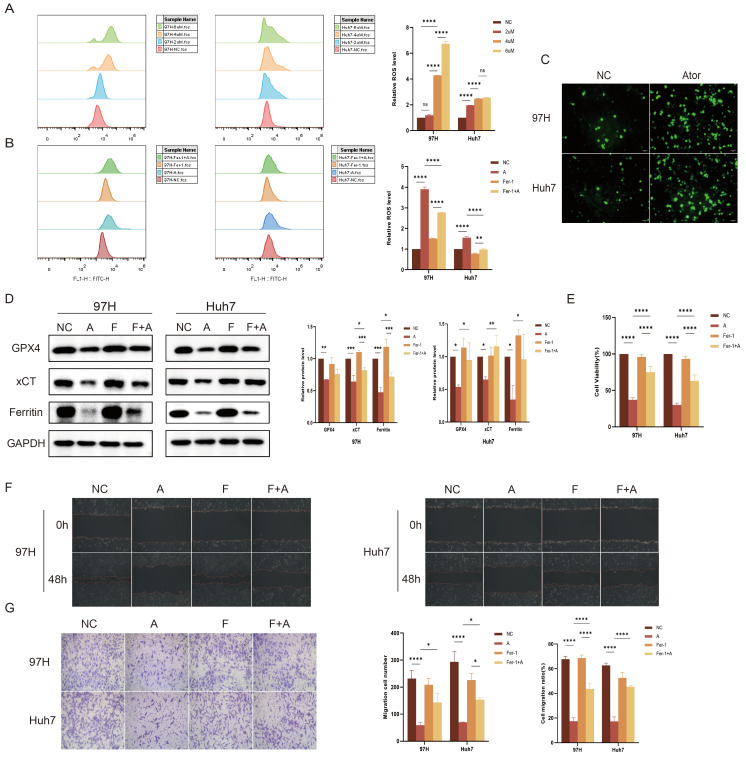
Atorvastatin induces ferroptosis in HCC cells. (**A**) The ROS levels of HCC cells treated with different concentrations of Atorvastatin are assessed by flow cytometry. (**B**) The ROS levels of HCC cells treated with 4 μM of Atorvastatin and 1 μM of Fer-1 are assessed by flow cytometry. (**C**) Observation of ROS fluorescence staining in each group. Scale bar: 50 μm. (**D**) Protein levels of GPX4, xCT, and Ferritin in HCC cells treated with 4 μM of Atorvastatin and 1 μM of Fer-1 are assessed using Western blotting. (**E**) Cell viability of HCC cells treated with 4 μM of Atorvastatin and 1 μM of Fer-1 is assessed using the CCK-8 assay. (**F**) The wound healing assay assesses the migration ability of HCC cells treated with 4 μM of Atorvastatin and 1 μM of Fer-1. Scale bar: 50 μm. (**G**) Cell invasion ability of HCC cells treated with 4 μM of Atorvastatin and 1 μM of Fer-1 determined by Transwell assay. Scale bar: 50 μm. Statistical results are presented. Data are presented as mean ± SD from three independent experiments. * *p* < 0.05, ** *p* < 0.01, *** *p* < 0.001, and **** *p* < 0.0001, ns means not significantcompared to NC group.

Ferroptosis is a form of regulated cell death primarily induced by the inactivation of the membrane lipid repair enzyme GPX4. This inactivation leads to the accumulation of ROS through Fenton reactions, ultimately disrupting essential proteins and resulting in cell death. Our findings indicate that an increase in Atorvastatin concentration correlates with a significant rise in ROS production in HCC cells (Figure 9A) and that the application of ferroptosis inhibitors (Fer-1) can effectively reduce ROS levels in the treatment group (Figure 9B). Furthermore, the fluorescence imaging of ROS following drug treatment in HCC cells further corroborated the above findings (Figure 9C). The results of Western blot analysis confirmed that Atorvastatin decreases the expression of ferroptosis-related proteins GPX4, xCT, and Ferritin in HCC cells, while the ferroptosis inhibitor Fer-1 significantly reversed this trend (Figure 9D). Additionally, the reduced cellular viability, migration, and invasion capacity induced by Atorvastatin can be reversed by Fer-1 (Figure 9E–G). Thus, we conclude that Atorvastatin induces ferroptosis in HCC cells by enhancing ROS production through the inhibition of GPX4.

### 3.12. Atorvastatin Exhibits an Anti-HCC Effect In Vivo

**Figure 10:**
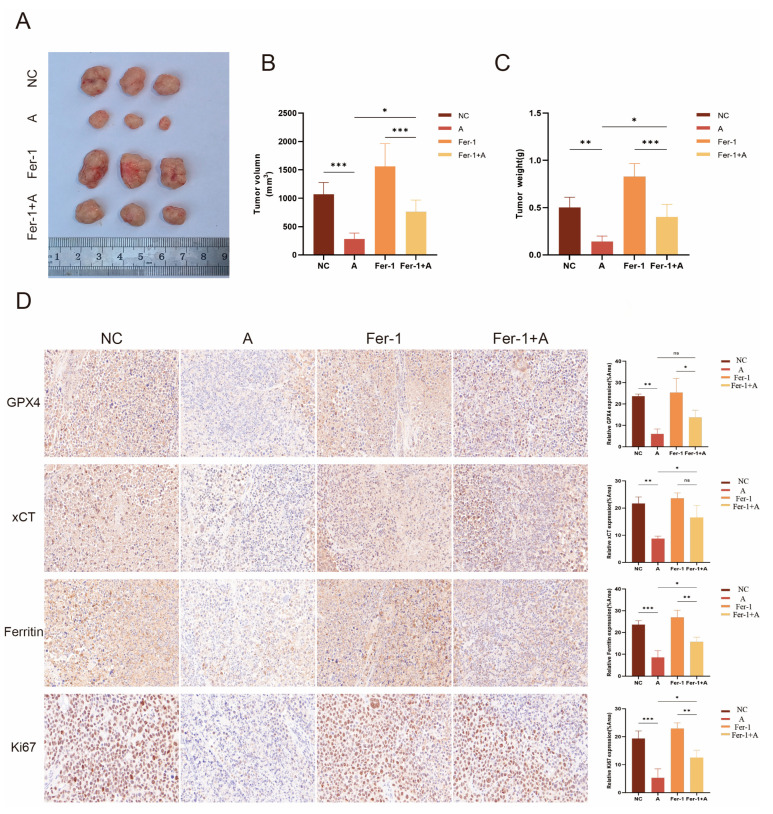
Atorvastatin exhibits an anti-HCC effect in vivo: (**A**) Images of subcutaneous tumor xenografts from nude mice treated with the same amount of DMSO, Atorvastatin (10 mg/kg), Fer-1 (1 mg/kg), Atorvastatin (10 mg/kg) + Fer-1 (1 mg/kg). (**B**) Tumor volume at the end of the experiment. (**C**) Tumor weight at the end of the experiment. (**D**) Immunohistochemical detection of GPX4, xCT, Ferritin, and Ki67 expression and quantitative analysis of staining intensity. Original magnification: 630×, scale bar: 20 μm. Statistical results are presented. All data are shown as the mean ± SD from three independent experiments. * *p* < 0.05, ** *p* < 0.01, and *** *p* < 0.001, ns means not significant compared to DMSO.

Based on the above in vitro findings, we further investigated the biological effects of Atorvastatin in vivo. Consistent with in vitro results, Atorvastatin significantly inhibited the growth of xenograft tumors. As shown in Figure 10A–C, the tumor volume and weight in the Atorvastatin treatment group were significantly reduced compared to the control group, whereas the inhibitory effect of Atorvastatin on tumor growth was blocked by Fer-1. The immunohistochemical staining intensity for Ki67, which can be used to assess the proliferative status of cells, was strongest in the control group and weakest in the Atorvastatin-treated group (Figure 10D). Finally, we assessed the levels of GPX4, xCT, and Ferritin in tumor tissue. As shown in Figure 10D, treatment with Atorvastatin resulted in decreased levels of GPX4, xCT, and Ferritin.

## 4. Discussion

Liver cancer remains a common and leading cause of cancer-related mortality [20]. HCC is the predominant type of liver cancer, accounting for approximately 80% [21]. The overall survival of HCC is poor, with a median survival time ranging from 6 to 10 months [22]. Due to the subtlety of early symptoms, nearly half of HCC cases are diagnosed at an advanced stage. Compared to localized HCC, advanced cases receive fewer curative treatments, resulting in lower survival rates [23]. Recent efforts have focused on the in-depth identification and study of early-stage HCC patients, revealing that biomarkers may provide accurate and valuable information for future personalized diagnosis and prognosis of HCC. Consequently, the urgent need for more effective biomarkers in the early detection and prognostic prediction of HCC has become increasingly evident [6]. In 2012, Berent et al. first introduced the term “ferroptosis” to describe a distinct form of iron-dependent regulated cell death driven by lipid peroxidation [24]. As research on ferroptosis advances, investigators have uncovered its close association with immune responses, development, aging, and tumorigenesis. There is evidence suggesting that ferroptosis can inhibit tumor development [25,26].

This study primarily utilized sequencing and clinical data from TCGA and ICGC to evaluate the prognostic relationship between ferroptosis-related genes and patients with HCC through bioinformatics approaches. First, we identified differentially expressed ferroptosis-related genes between normal and HCC tissues based on the HCC dataset from TCGA. Subsequently, various types of regression analysis were employed to determine four ferroptosis-related prognostic genes as key biomarkers. Kaplan–Meier analyses showed a significant difference in OS between the two risk groups and a better prognosis in the low-risk group. Survival status distribution, PCA analysis, and ROS analyses indicated that the identified prognostic key genes could effectively stratify patients into high-risk and low-risk groups, as well as predict the overall survival of HCC patients. In summary, our work provides valuable tools for the diagnosis and prognosis of HCC, facilitating advanced personalized treatment for patients and improving the efficacy of disease management.

The four prognostic-related genes identified in this study were significantly upregulated in HCC tissues, correlated with the infiltration of multiple immune cell subtypes, and demonstrated poor survival outcomes. Furthermore, these genes have been investigated in various cancer types and are implicated in tumor-associated pathways. G6PD is the rate-limiting enzyme of the pentose phosphate pathway (PPP), responsible for converting G6P into 6-phosphogluconolactone (6PGL) [27]. Studies have revealed that G6PD is aberrantly upregulated in various types of cancer, impacting tumor-associated biological processes such as cell cycle regulation, DNA synthesis, and repair, thereby creating conditions conducive to tumorigenesis and progression [28,29,30]. G6PD inhibits ferroptosis in HCC cells, while its knockdown reduces tumor volume and weight in vivo [31]. To date, numerous studies have established the critical role of SLC7A11-mediated cystine uptake in suppressing ferroptosis and maintaining cell survival under conditions of oxidative stress [24,32,33]. Moreover, SLC7A11 has emerged as a central hub linking ferroptosis and tumor suppression functions. The loss of tumor suppressor genes, mutations in oncogenes, or the overexpression of pro-tumor proteins elevates levels of SLC7A11 in cancer, resulting in the inhibition of ferroptosis and enhanced tumor progression [32,33,34,35]. *MYCN* is a well-known oncogene that is overexpressed in various malignant tumors, including neuroblastoma, rhabdomyosarcoma, medulloblastoma, and small-cell lung cancer [36,37,38,39]. Numerous studies have indicated that *MYCN* functions as a key gene influencing ferroptosis and is involved in the initiation and progression of cancer [40,41]. Molecular motors play a critical role in force generation, migration, and intracellular transport. KIF20A, a motor protein belonging to the dynein family, exhibits a negative correlation with clinical outcomes in cancer [42]. Previous reports have indicated that KIF20A is highly upregulated in various cancer cell types, including pancreatic cancer, melanoma, bladder cancer, and HCC [43,44,45,46,47,48]. KIF20A can inhibit ferroptosis in colorectal cancer cells, thereby reducing their sensitivity to oxaliplatin [49]. Based on the aforementioned findings, we reasonably hypothesize that promoting ferroptosis in HCC cells may be achieved by inhibiting these ferroptosis-related oncogenes. Consequently, we further explored and screened potential therapeutic agents with efficacy in HCC using the CMap database.

Currently, chemotherapy is a common approach for patients with advanced HCC; however, it is associated with severe adverse effects. The development of new drugs is a costly endeavor, often requiring several decades and expenditures exceeding hundreds of millions of dollars [50]. Therefore, the repurposing of drugs with established indications for the development of anticancer therapies has become increasingly important. These drugs, which typically have fewer adverse effects, can enter clinical use more rapidly and at a lower cost. Consequently, drug reutilization continues to be explored and widely applied [51]. The CMap database is a gene expression-based drug research platform that links genes, drugs, and diseases through extensive experiments conducted across a multitude of cell lines [52]. Through bioinformatic analyses and exploration of the CMap database, we identified key genes associated with HCC and the candidate therapeutic agent Atorvastatin, aimed at inducing ferroptosis in HCC cells and treating the disease. The CCK-8 assay, scratch wound healing assay, and invasion assay indicated that Atorvastatin significantly induces cell death and inhibits cell proliferation and migration. These findings support our hypothesis that Atorvastatin may be an effective therapeutic agent for HCC. Numerous studies have demonstrated that ferroptosis can be assessed through various methods, including the evaluation of cell viability, the expression of key ferroptosis-related genes such as xCT and GPX4, and the measurement of ROS levels [53]. Subsequent experiments revealed that Atorvastatin increases the levels of ROS in HCC cells while decreasing the expression of genes that negatively regulate ferroptosis. This effect was reversible by the ferroptosis inhibitor Fer-1. Consistent results were observed in follow-up animal studies, confirming that Atorvastatin induces ferroptosis in HCC cells and inhibits tumor progression.

At present, triggering ferroptosis appears to be one of the most promising strategies for inhibiting tumors and treating drug-resistant cancer cells. Four distinct mechanisms that induce ferroptosis have been identified: Class I, which acts by depleting glutathione (GSH); Class II, which inactivates GPX4; Class III, which consumes GPX4 protein and depletes CoQ10; and Class IV induces lipid peroxidation [15]. Various systemic agents, including targeted therapies, chemotherapeutics, lipid-lowering agents, and anti-inflammatory drugs, have been identified as ferroptosis inducers (FINs) with tumor-suppressive capabilities [54]. Statins are a class of lipid-lowering agents, including Atorvastatin, lovastatin, and simvastatin. These agents selectively and competitively inhibit hydroxy-3-methylglutaryl coenzyme A (HMG-CoA) reductase, suppressing cholesterol biosynthesis. The downregulation of GPX4 expression is the primary mechanism involved in statin-mediated ferroptosis inhibition. This occurs because HMG-CoA reductase inhibition reduces mevalonate (MVA) production, which in turn decreases downstream isoprenoid metabolites such as isopentenyl pyrophosphate (IPP), which participated in GPX4 synthesis and maturation [55]. GPX4, a key regulator of ferroptosis, exerts cytoprotective effects by detoxifying phospholipid hydroperoxides (PLOOHs). The loss of GPX4 function disrupts this protective mechanism, leading to iron-dependent lipid peroxidation accumulation and subsequent cell death [56]. Additionally, a reduction in IPP, the precursor of coenzyme Q10 (CoQ10), enhances lipid peroxidation capacity [57]. Notably, multiple preclinical studies have demonstrated statin-induced ferroptosis in cancer cells through this pathway [55,58]. Simvastatin inhibits the expression of 3-hydroxy-3-methylglutaryl coenzyme A reductase (HMGCR), downregulating the mevalonate pathway and GPX4, thereby inducing ferroptosis in cancer cells [59]. Elakkad et al. demonstrated that simvastatin-loaded cubosomes significantly reduced the levels of anti-apoptotic Bcl-2 protein, GSH, and GPX4 in breast cancer cells while markedly increasing ROS and lipid peroxide levels [60]. In general, the antitumor effects of statins have been gradually recognized; however, further research is warranted to delineate the underlying mechanisms and to optimize their clinical application as anticancer agents.

The principal agent of this study, Atorvastatin, showed in prior research the ability to inhibit myocyte viability in a dose-dependent manner, accompanied by a significant increase in intracellular iron ions, ROS, and lipid peroxidation [61]. It was reported to modulate ferroptosis in rat cardiomyocytes (H9C2 cells) by activating SMAD7 expression to regulate the hepcidin/ferroportin 1 (FPN1) axis, promoting iron ion efflux in H9C2 cells [62]. In adipose tissue, the Atorvastatin-treated group showed increased levels of ROS, malondialdehyde (MDA) and Fe^2^⁺, accompanied by the decreased expression of GSH and GPX4 [63]. These changes are thought to induce ferroptosis by reducing the production of geranylgeranyl pyrophosphate (GGPP). Atorvastatin has demonstrated emerging research significance in oncology: Combined with gemcitabine, it suppresses cholangiocarcinoma cell proliferation and promotes apoptosis by inhibiting YAP nuclear translocation and TEAD transcriptional activation, exhibiting enhanced anticancer efficacy [64]. It induces growth inhibition and G0/G1 phase cell cycle arrest, leading to senescence in HCC cells [65]. Through autophagy activation and PERK/ATF4/CHOP signaling pathway stimulation, it triggers colorectal cancer cell apoptosis while synergistically enhancing 5-FU-mediated cytotoxic effects [66]. Regarding the research of targeting cancer ferroptosis, in vitro and in vivo experiments demonstrated that Atorvastatin can induce ferroptosis and sensitize radioresistant HNSCC cells to radiation, thereby promoting cell death sensitivity [67]. Research regarding the utilization of Atorvastatin to target ferroptosis for cancer treatment remains limited. The specific mechanisms through which Atorvastatin induces alterations in the characteristic molecules and indices associated with ferroptosis remain to be elucidated. Future research endeavors are expected to concentrate on exploring the intricate relationships among Atorvastatin, ferroptosis, and HCC.

Although our current study successfully established predictive indicators for HCC and identified Atorvastatin as a critical drug repurposing candidate with anticancer potential, several limitations and constraints should be acknowledged: (1) Data Limitations: While public databases offer accessibility and broad applicability, incomplete clinical annotations and the absence of therapeutic context may introduce bias. Future studies should integrate multicenter clinical data to enhance model robustness and conclusion reliability; (2) Drug Screening Constraints: Due to resource limitations and prioritized focus on lipid-lowering agents (given their pre-established safety profiles), the systematic validation of other candidate drugs was regrettably not feasible. In future investigations, this aspect will be addressed through expanded screening protocols and rigorous prioritization processes; (3) Translational Considerations: As a lipid-lowering agent, Atorvastatin modulates lipid levels and other physiological functions in the human body. The translation from experimental findings to clinical applications requires meticulous dose optimization, avoiding high-dose toxicity while ensuring therapeutic efficacy, and the continuous monitoring of lipid profiles and vital organ functions in experimental models to guarantee drug safety.

In summary, this study systematically identified core ferroptosis-related genes to construct and validate a prognostic model. Beyond predicting patient survival, our goal was to discover potential ferroptosis-targeting therapeutics for HCC. Through drug screening in the CMap database based on HCC-ferroptosis gene signatures, Atorvastatin was prioritized and experimentally validated. These achievements provide novel prognostic biomarkers for HCC and lay the groundwork for targeted therapy.

## 5. Conclusions

This study identified four ferroptosis-related prognostic genes in HCC and validated their potential as novel biomarkers for the personalized treatment of HCC patients. We screened for potential drugs targeting ferroptosis, identifying the compound Atorvastatin, which was found to inhibit cancer cell growth and migration by inducing ferroptosis in HCC. Our study enhances the understanding of the relationship between ferroptosis and cancer therapy, providing new insights for the development of anti-tumor agents based on ferroptosis mechanisms.

## Figures and Tables

**Table 1:** Clinicopathological parameters of HCC patients used in this study.

Variables	TCGA (*n* = 326)	ICGC (*n* = 258)
Age, *n* (%)		
<65	201 (61.66)	91 (35.27)
≥65	125 (38.34)	167 (64.73)
Gender, *n* (%)		
Female	101 (30.98)	214 (82.95)
Male	225 (69.02)	44 (17.05)
OS days	879	803
Survival status, *n* (%)		
Alive	215 (65.95)	68 (26.36)
Dead	111 (34.05)	190 (73.64)
Stage, *n* (%)		
I	164 (50.30)	40 (15.51)
II	78 (23.93)	117 (45.35)
III	81 (24.85)	78 (30.23)
IV	3 (0.92)	23 (8.91)
T, *n* (%)		
T1	165 (50.61)	NA
T2	79 (24.23)	
T3	72 (22.09)	
T4	10 (3.07)	
M, *n* (%)		
Mx	74 (22.70)	NA
M0	249 (76.38)	
M1	3 (0.92)	
N, *n* (%)		
Nx	79 (24.23)	NA
N0	244 (74.85)	
N1	3 (0.92)	

**Table 2:** Top 10 search results from CMap for two-risk HCC groups.

Number	Name	Score	FDR
1	PHF2	−1.88	15.65
2	BRSK2	−1.87	15.65
3	tiabendazole	−1.86	15.65
4	reversine	−1.84	15.65
5	etoposide	−1.83	15.65
6	PON3	−1.83	15.65
7	HEBP1	−1.82	15.65
8	TWS-119	−1.80	15.65
9	ZNF385B	−1.80	15.65
10	GPR64	−1.80	15.65

**Table 3:** Top 10 search results from CMap for ferroptosis.

Number	Name	Score	FDR
1	azacitidine	−1.99	15.65
2	anisomycin	−1.99	15.65
3	heliomycin	−1.92	15.65
4	BRAF	−1.92	15.65
5	clofarabine	−1.90	15.65
6	etoposide	−1.89	15.65
7	pralatrexate	−1.88	15.65
8	CRH	−1.87	15.35
9	GPR146	−1.87	15.35
10	eltrombopag	−1.86	15.35

## Data Availability

Publicly available datasets were analyzed in this study. These data can be found here: http://www.cancergenome.nih.gov/, https://www.ncbi.nlm.nih.gov/gds/, https://dcc.icgc.org/, http://www.zhounan.org/ferrdb/index.html (accessed on 23 May 2024).

## Supplementary Materials

The following supporting information can be downloaded at https://www.mdpi.com/article/10.3390/cimb47030201/s1.
