# Supplementary figures and images for: A Novel Ferroptosis-Related Gene Prognosis Signature and Identifying Atorvastatin as a Potential Therapeutic Agent for Hepatocellular Carcinoma
Source: Curr Issues Mol Biol. 2025 Mar 18;47(3):201. doi: 10.3390/cimb47030201 (PMC11940908; doi:10.3390/cimb47030201)

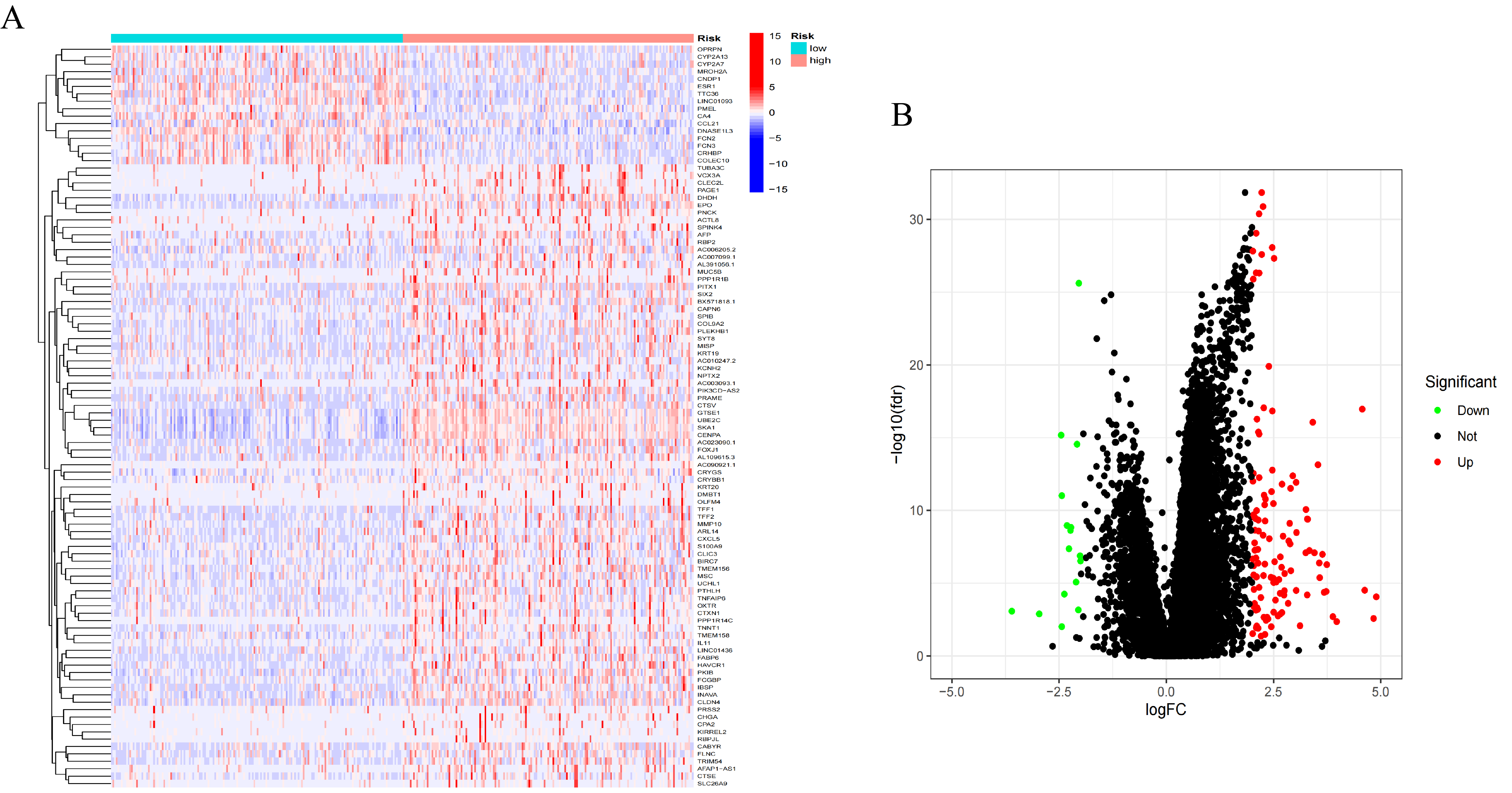

Supplement: Supplementary file 1 [file cimb-47-00201-s001.zip › Figure S1.tif]
